# A Rare and Potentially Catastrophic Infection: Primary Intestinal Aspergillosis—Case Report in an HIV Patient

**DOI:** 10.1155/2018/3269847

**Published:** 2018-07-08

**Authors:** Cátia Dias, Filipa Duarte-Ribeiro, Sara Pipa, Margarida Mota

**Affiliations:** Department of Internal Medicine, Centro Hospitalar Vila Nova de Gaia/Espinho, Espinho, Portugal

## Abstract

Aspergillus species are ubiquitous in nature; however, infection is uncommon, except in immunocompromised or immunosuppressed hosts. We present the case of a 71-year-old woman with a history of human immunodeficiency virus infection who presented with fever, weight loss, and diarrhea, posteriorly diagnosed with intestinal aspergillosis after examination of a segmental enterectomy piece. The diagnosis was made postmortem once the patient died after fast and progressive deterioration in the postoperative period.

## 1. Introduction

Aspergillosis is the disease caused by species of *Aspergillus* spp. These are ubiquitous in nature and their inhalation is common in the general population. However, tissue invasion and consequent infection are uncommon and usually involves some degree of immunosuppression, which alters the immune response to inhaled species. We present the case of a 71-year-old woman with a history of human immunodeficiency virus infection who was diagnosed postmortem with intestinal aspergillosis after histological examination of a segmental enterectomy piece.

## 2. Case Description

**Figure 1:**
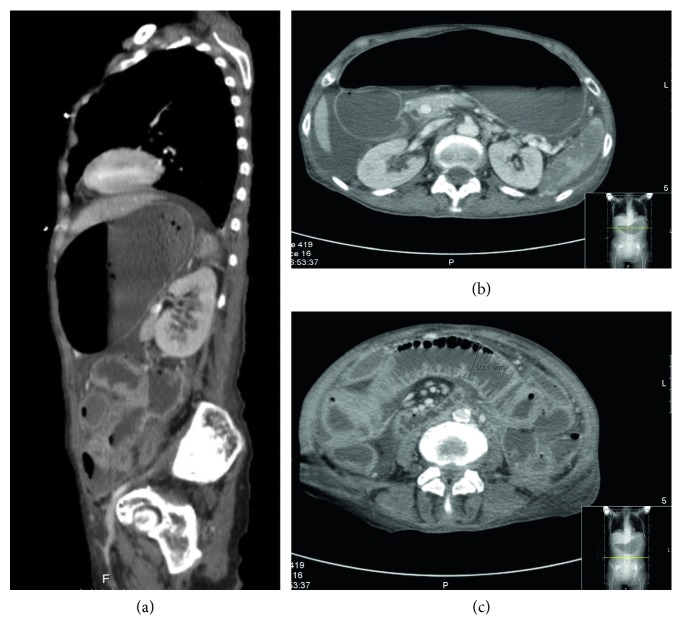
Thoracic, abdominal, and pelvic CT from the day of admission in (a) sagittal and (b, c) transverse planes.

A 71-year-old woman with a past medical history of uterine cancer 25 years before, herpes-zoster infection two years before, recent diagnosis of human immunodeficiency virus (HIV) infection, and cervical adenopathies under investigation presented at the first medical appointment at the Infectious Diseases Unit referring a 3-week history of fever, weight loss of 20 kg, and hemoptoic cough, as well as diarrhea with one year of evolution. On physical examination, she was cachectic and weak, had axillary temperature of 38°C, blood pressure of 112/80 mmHg, respiration rate of 40 per minute, heart rate of 142 beats per minute, and oxygen saturation of 95% in room air. She also presented with pain and tenderness at the palpation of the hypogastric region, and during the consultation, she presented cardiorespiratory arrest. Advanced life support with favorable response was performed, and she was subsequently transferred to the emergency room where it was necessary to initiate aminergic support and proceed to orotracheal intubation and mechanical invasive ventilation. The complementary diagnostic exams revealed white blood cell count 14,740/*μ*L with absolute neutrophil count 13,180/*μ*L (89.4%) and absolute lymphocyte count 970/*μ*L (6.6%) with 113 CD4+/*μ*L cells, hemoglobin level 11.3 g/d, and platelet count 2,33,000/*μ*L. She presented with blood creatinine 1.34 mg/dL, pancreatic amylase 222 U/L (4 times above the upper limit of normal), pancreatic lipase 174 U/L (3 times above the upper limit of normal), and seric lactates 6.5 mmol/L. Viral load of HIV by polymerase chain reaction was 2,330,220 copies/mL. Thoracic, abdominal, and pelvic computed tomography (CT) revealed pneumoperitoneum, peritonitis, diffuse parietal thickening, and dilatation of the intestinal loops of the jejunum with splenic infarction (Figure 1).

An emerging surgery exploratory laparotomy was performed having been found enteric peritonites of the large cavity, occlusion with transition point at the level of the distal jejunum and poor perfusion, and thickening of the distal loops and perforation at the level of the distal ileum. Segmental enterectomy of approximately 35 cm of jejunum/ileum has been performed, including ischemic loop and ileal perforation. In the postoperative period, she was admitted to the intensive care unit, and broad-spectrum antibiotic therapy has been initiated. Nevertheless, she evolved with progressive clinical deterioration with increasing need of amines and without favorable hemodynamic response. The patient passed away the same day. The histological examination of the segmental enterectomy piece and available postmortem revealed acute transmural inflammation of the enteric wall, with suppuration and necrosis and extensive lesions of acute fibrinoexudative serositis with fungal hyphae of the *Aspergillus* spp. type. There were no images of angioinvasion, epithelioid granulomas, or signs of malignancy. The culture of peritoneal fluid obtained during surgery revealed polybacterial and fungal overinfection with *Enterococcus faecium*, *Escherichia coli*, and *Candida albicans*.

## 3. Discussion

Aspergillosis may refer to several types of diseases such as allergic disease, invasion of the upper or lower airways, skin infection, or extrapulmonary disease, which may be due to tissue invasion and hematogenous dissemination, which can lead to disseminated infection. Invasive aspergillosis (IA) occurs almost exclusively in situations where cellular immunity is severely compromised. In fact, when immunity is intact, *Aspergillus* conidia and hyphae are inhaled but destroyed by alveolar macrophages and polymorphonuclear leukocytes. When there is a qualitative or quantitative defect of these cells, there may be angioinvasive growth with hemorrhage, thrombosis, hypoxia, necrosis, and dissemination [1]. The majority of cases of IA are described in patients submitted to allogeneic hematopoietic cell transplant or solid organ transplant, those with severe and prolonged neutropenia, and those in need of high doses of glucocorticoids or immunosuppressive regimens [2]. HIV infection, although infrequent, is one of the risk factors for IA, being present in approximately 4% of the cases of IA described [2], even though IA occurs in less than 1% of HIV-infected patients [3, 4].

In this group of patients, IA has a high morbidity and mortality, with fatality rates around 86% [2, 5]. The risk of occurrence of IA increases with highest degree of immunosuppression or in those with other risk factors for invasive disease, such as neutropenia or use of corticoids [6]. The incidence is higher in patients with CD4+ cells below 100/*μ*L and with one or more opportunistic diseases (acquired immunodeficiency syndrome (AIDS)-defining) [6]. However, in the highly active antiretroviral therapy (HAART) era, this paradigm has changed, with more cases being identified in patients with CD4+ cells higher then 100/*μ*L [7]. It should be noted that in the present case, none of the other risk factors presented above were present, with the exception of low CD4+ cells.

Most cases of invasive aspergillosis occur in the lungs and respiratory tract, and patients with HIV infection are no exception (approximately 61.2% of the cases). Cases of intestinal IA described in the literature are extremely rare, constituting only isolated clinical cases. Although other routes of entry to IA other than pulmonary are only speculative, it is thought that in the intestine, the invasion may be through local inoculation and there are, as in the case of the patient presented, some cases described in patients with proven intestinal IA but without pulmonary disease [8–10]. Intestinal aspergillosis may manifest as enterocolitis, appendicitis, colonic ulcers, abdominal pain, and gastrointestinal bleeding.

The diagnosis of IA is considered “proven” when the hyphae of *Aspergillus* spp. are observed together with evidence of tissue injury (as presented in this case), or when a positive culture for *Aspergillus* spp. is obtained from a specimen obtained by sterile procedure and from a normally sterile site [11]. Whenever possible, microbiological cultures and histology should be obtained; however, isolated positive cultures may only be related to contamination of the samples.

## 4. Conclusion

Although very rare, patients with HIV infection/AIDS are susceptible to *Aspergillus* infection, which can lead to disseminated disease with high rates of morbidity and mortality, with most cases of extrapulmonary infections being documented postmortem. This case emphasizes the importance of early consideration of this differential diagnosis when approaching HIV-infected patients with symptoms similar to those of the patient under discussion.
